# How do people with physical/mobility disabilities benefit from a telehealth exercise program? A qualitative analysis

**DOI:** 10.3389/fresc.2022.932470

**Published:** 2022-07-25

**Authors:** Jereme D. Wilroy, Yumi Kim, Byron Lai, Nataliya Ivankova, Ivan Herbey, Tanvee Sinha, James H. Rimmer

**Affiliations:** ^1^Department of Physical Medicine and Rehabilitation, The University of Alabama at Birmingham, Birmingham, AL, United States; ^2^UAB-Lakeshore Foundation Research Collaborative, Birmingham, AL, United States; ^3^Division of Pediatric Rehabilitation Medicine, Department of Pediatrics, The University of Alabama at Birmingham, Birmingham, AL, United States; ^4^Department of Health Services Administration, The University of Alabama at Birmingham, Birmingham, AL, United States; ^5^Department of Surgery, The University of Alabama at Birmingham, Birmingham, AL, United States; ^6^Dean's Office, The University of Alabama at Birmingham, Birmingham, AL, United States

**Keywords:** disability, exercise, telehealth, mobile health (mhealth), qualitative

## Abstract

People with neurological and physical disabilities (PWD) experience a myriad of secondary and chronic health conditions, thus, reducing their participation and quality of life. A telehealth exercise program could provide a convenient opportunity for improving health in this population. To describe participants' perceived benefits of a telehealth physical activity program among PWD, we conducted semi-structured interviews with 30 study participants after completing the 24-week program SUPER-HEALTH (Scale-Up Project Evaluating Responsiveness to Home Exercise and Lifestyle TeleHealth). Interview data were recorded, transcribed verbatim, and analyzed using inductive thematic analysis. The mean age of the sample was 51 ± 13 years, the primary disability was Multiple Sclerosis, and there were nine men (30%) and 21 (70%) women. Inductive thematic analysis resulted in four themes that include the following: (1) improved health and function, (2) increased activity participation, (3) improved psychosocial health, and (4) optimized performance and benefits. These preliminary findings provided support for the use of a home exercise program and recommendations to improve it to enhance benefits among PWD.

## Introduction

People with disabilities (PWD) are highly susceptible to secondary health conditions, including osteoporosis, osteoarthritis, decreased balance, strength, endurance, fitness, flexibility, increased spasticity, weight problems, and depression (1–4). These secondary health conditions are exacerbated by a physically inactive lifestyle prevalent in this population, which has been linked to poor health outcomes (5, 6). Compared to people without disabilities, PWD is three times more likely to experience a stroke or heart attack (7–9). Additionally, this places a further burden on the healthcare system (8, 10).

The health needs of PWD are extensive and drive the need for health promotion strategies and catered rehabilitative care in and outside of medical institutions (11). While some preliminary exercise interventions have shown to improve functional motor recovery and overall health in patients needing neurological care (12), many current rehabilitation models do not have a systematic prescription to optimize exercise maintenance in the long run. Care is prioritized in the acute period after the neurological injury/diagnosis and is focused on the recovery of ADL skills and basic mobility rather than improving the sedentary lifestyle (11). Unfortunately, this sedentary lifestyle can be attributed to the many barriers encountered by this population at every level of the socioecological model, including the intrapersonal level (e.g., low self-efficacy); interpersonal level (e.g., low social support), organizational level (e.g., lack of programming or trained personnel), community (e.g., inaccessible parks), and policy (e.g., local transportation) (13). If patients had means of accessing care acutely and in the long term, medical personnel could emphasize not only restoring ADLs in their rehabilitation, but also promoting exercise and physical mobility to improve quality of life and reduce further complications.

To address these barriers, researchers in rehabilitation sciences and health education have adapted health interventions to allow for online delivery and two-way audio-visual communication, also known as telehealth (14). Studies have shown that telehealth has proven to be effective for the rehabilitative management of patients because of reduced health care expenses, easy access to care (without any transportation), and less disruption to care (15). Allowing participants to engage with health-enhancing programs, and as a physical activity-based intervention, from the comfort of their own home is a notable benefit in convenience for patients.

To evaluate whether a telehealth exercise program can increase physical activity and improve functional outcomes and quality of life in PWD, a home-based exercise program was developed, the SUPER-HEALTH (Scale-Up Project Evaluation Readiness to Home Exercise and Lifestyle Tele-Health). This program was delivered *via* a mobile application (16) to provide the convenience of completing the program at home, which removes the barriers of transportation and inaccessible facilities, in addition to lack of knowledge and adapted exercises. The SUPER-HEALTH program addresses this issue by providing PWD with an online program that was modified from an on-site, evidence-based exercise intervention called Movement-to-Music (M2M). The current study aimed to explore participants' perceptions of potential benefits from a home-exercise program, with a specific focus on benefits related to physical health and function, participation, and psychosocial health. The second aim was to describe recommendations to enhance the benefits that could be received from the program.

## Methods

### Design

This study used a qualitative research design involving semi-structured interviews with purposefully selected individuals among a cohort of PWD who completed an RCT of physical activity. This study was approved by the institutional review board at the university, and participants provided verbal consent before participation.

#### SUPER-HEALTH program

The M2M is set to music to provide greater enjoyment from an exercise routine and includes aerobic and strength training (17). The structure of each M2M session includes a range of motion, muscle strength, balance, cardiorespiratory endurance, and cooldown. The application releases a new pre-recorded M2M exercise video each week for the participants, so they can use it to meet their weekly exercise goal. When a new routine is introduced, the routine is guided by an M2M instructor who provides verbal instruction and explains each movement pattern. The following week, the same routine is performed by a person with a similar disability but with no verbal instruction, and a new guided routine is also delivered. The routines delivered to participants were choreographed to music and designed for three functional groups: those able to stand, those seated only, and those with hemiparesis. The M2M videos incorporate ‘public domain' music to avoid copyright issues.

SUPER-HEALTH includes a 12-week adoption phase, a 12-week transition phase, and a follow-up completed at 48 weeks. The intervention includes a prescription of 48 min of exercise video content on the first week, and this amount increases each week, with 150 min delivered at week 24. Each participant received a Fitbit and a tablet with a study app. Research staff monitored participants' Fitbit and tablet activity each week and provided a coaching support call for participants with no activity. More details of the study are reported elsewhere (16).

### Participants

Eligibility criteria for SUPER-HEALTH included: (a) self-report of a physical disability or mobility impairment, (b) 18 to 74 years of age, (c) not enrolled in a structured exercise program over the past 6 months, (d) can use upper, lower, or both sets of extremities to exercise, € can converse and read English, (f) medically stable to perform the home exercise as determined by their physician, and (g) wireless internet in the home. For the current study exploring program experiences, SUPER-HEALTH participants were selected for an interview after completing 24 weeks using a purposeful sampling strategy to promote diversity among interviewed participants. Participants were selected based on the following characteristics: functional level during exercise (seated, standing, and hemiparesis), gender, and race. The research team recorded this information before each interview to ensure that the sampling strategy was executed. This project aimed to recruit a convenience sample of 30 participants who had completed at least 24 weeks (primary endpoint) of the SUPER-HEALTH study.

### Procedures

Participants who agreed to be interviewed were scheduled, and all interviews were completed over the phone. The interviews were semi-structured, with a max duration of 30 min. Interview questions focused on understanding participants' perceived benefits of the program regarding their physical and functional health, mental health, social health, and any other perceived benefits. Questions also included participant suggestions for enhancing perceived benefits in future telehealth exercise programs. Sample questions are displayed in Box 1.

Box 1 **Sample interview questions**

• Please tell me about your experience with the SUPER-HEALTH program.

• What benefits have you seen when exercising with the program?

• How do you feel when you exercise with the program?

• Describe to me some positive experiences of the program.

• What did you enjoy most about this program?

• Describe to me some negative experiences or issues you experienced with the program.

• What did you least like about this program?

• What did you find useful about this program? Why?

• What did you not find useful about this program? Why?

• How do you think this program helps you exercise more?

• What suggestions do you for improving the program?

#### Research team

For this study, the research team included six PhD-level academic researchers with two experts in qualitative research methods (NI and IH) and four researchers with a background in rehabilitation science (JW, JR, YK, and BL). One researcher (JW) has a disability and completed the interviews as part of a KL2 Mentored Career Development award. Another researcher (JR) was the mentor for the award and principal investigator of the exercise trial, from which the participants were interviewed.

### Analysis

Demographics and disability characteristics were reported to describe the sample. Coding and analysis were guided by the Braun and Clarke 6-step thematic analysis (inductive) approach (17). First, interviews were transcribed by a professional transcription company and verified for accuracy by three analysts (JW, YK, and IH). Second, the analysts coded the data separately to generate an initial set of codes. Third, the analysts met to compare and contrast codes and search for themes (categories that represent the codes). Fourth, the analysts generated an initial set of themes into a small number of themes that were deemed saturated (i.e., sufficiently supported by participant quotes and codes). Fifth, they narrowed the themes into higher-level groupings (2nd tiered themes). Sixth, the themes were documented and reported. The iterative data analysis and discussion processes contributed to achieving trustworthiness between the two analysts (18). All analysts were not involved in conducting the intervention and had a background in adapted physical activity or rehabilitation science (JW and YK) and qualitative research (IH). In addition, the “critical friends” were involved to ensure an appropriate research process and weight on the interpretation of the relevance of themes (19). The critical friends in this study have been prolific in the field of rehabilitation research (BL) and qualitative research (NI), respectively.

## Results

**Figure 1:**
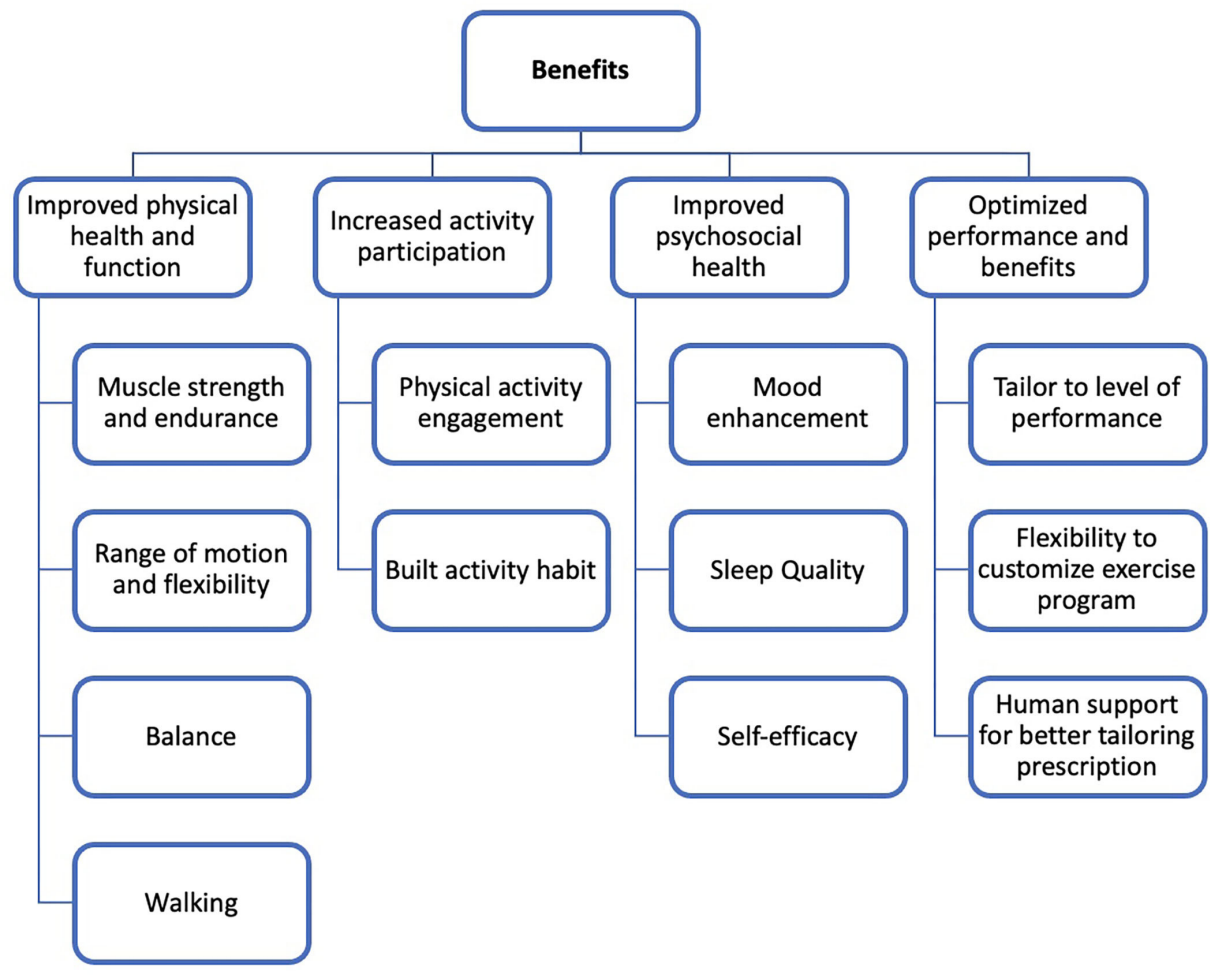
Perceived benefits to SUPER-HEALTH among people with disabilities.

The mean age of the sample was 51 ± 13 years. The overall samples predominantly consisted of women (*n* = 21/30, 70%) and Caucasians (*n* = 21/30, 70%). Disabilities included eight with multiple sclerosis (26.7%), three Parkinson's Disease (10%), three spinal cord injury (10%), three spina bifida (10%), three stroke (10%), and six with spinal disorders (20%), such as scoliosis. Lastly, participants were selected based on a functional level for exercise routines, including 16 who were able to exercise standing, 12 sitting, and two with only one side of their body (i.e., hemiparesis). All the contacted participants agreed to complete the interview. Table 1 displays all themes, sub themes, and illustrative quotes and Figure 1 depicts the organization of themes and sub themes.

**Table 1:** Themes, subthemes, and illustrative quotes.

Improved physical health and function	
Muscle strength/endurance	Well, it was actually good to get, um, my motor skills and actually using my legs and doing things like that. It- it helps out. I don't feel more- as much of a need to use a cane if I feel strong- my legs are stronger if that makes sense? (ID 166) I did notice a difference in my muscles getting stronger and stuff, so that was a reward, rewarding thing (ID 188). Yeah, definitely, weight loss. And I've actually, you know, had more strength in my legs, you know, when I wasn't able to walk. Like certain exercises that was on the video, I've definitely seen like an improvement, you know, in the strength on like the left side of my body where the weakness was (ID 379). Strengthening my core. That's something that we're really trying to focus on because it takes the pressure off of the injury and just helps with pain. So, pain decreases (ID 419). My legs feel stronger if I actually stand up and do the exercises. I stand up and do them, so I just want my legs to be stronger. Because that's my issue with MS is my legs go out (ID 445). My arms are getting stronger from the program. The particular one that I was doing is they focus on the top part of the body (ID 448).
Range of motion/Flexibility	Well, my muscles are not as tight when I exercise and that helps reduce pain if they're not, um, all tensed up. It, it makes it hurt, but with the ex- with the program, it helps me to exercise. So that helps with the, um, cervical and, uh, lumbar pain (ID 271). It keeps my back, and my arms, and my shoulders lose (ID 292). I can tell a difference. It keeps my joints and everything moving and very fluid and all. Because sometimes when I wake up, I'm really stiff, but I can tell a difference doing all that (ID 334). The stretching really helps because you get really stiff. It doesn't take much for me to get stiff (ID 334).
Balance	The range of motion exercises has helped more than anything because I do have a high level of balance issues and dizziness, and when I'm driving, the turn of the head to look this way and that way for cars, the range of motion video has helped just tremendously (ID 327). I think I'm getting around a little better. I think it [the SUPER-HEALTH program] helps with balance (ID 334). It's just the movement increases, your ability to move and agility is there and that kind of stuff. Helps me to be me (ID 305).
Walking	I just have more energy, and I'm like, I feel like my walking, my gait's better (ID 198) I've enjoyed this program, it's um, I've lost weight, I'm stronger, I, since I've been in the two studies [TEAMS – another tele exercise program & SUPER-HEALTH] that I've been in, I no longer walk with a cane, um, I don't drag my right leg as much as I used to, so I see physical improvement (ID 228). I'm able to control my breathing, and I'm able to get a little stronger and move around more (ID 443).
Increased activity participation	
Physical activity engagement	Well, it [physical activity level] changed, it did change, um, it [the SUPER-HEALTH program] sort of just made me more aware of what I was missing. Like I said, you know coming from being like, from the time I was like what 10 years old, to the end of college, I was just constantly an athlete. And that was like, the main thing I did. And then when I graduated and got working, and realized work was eating my life, and I wasn't doing anything physical…I think it [the SUPER-HEALTH program] did change things overall. You know, like when I do have time, I do look for things that I can do physically. So, I think that sort of highlighted that (ID 58). I mean, it [the SUPER-HEALTH program] made me get up and move and exercise (ID 198). It [the SUPER-HEALTH program], it did help me to move forward and becoming more active… just being able to be, um, with mo- to be able to be active during the daytime that helped with not working, being more active during the daytime, going for walks and, and being outside and, and, uh, that helped a lot (ID 258). Um, it has definitely got me up and doing cardiovascular and walking and counting my steps (ID 287) It's [the SUPER-HEALTH program] got my heart rate up a pretty good bit. I'm not just sitting around doing nothing. So, I know how to get up and get going every other day to get my workouts done (ID 292). [What maybe value did this program add to your exercising?] Anything to keep me moving, keep me doing stuff (ID 305). Yeah, because even the weights, I have them sitting by the couch so a lot of time I can just pick them up. And I'm more determined to kind of work on my upper arms so I try to pick them up a lot while I'm sitting looking at TV and just work with those, too (ID 384).
	I would say I can exercise more by doing it, exercise more three times a week and exercise longer than I usually do (ID 443). Plus, it is easier, it seems to me, to try to stay more active. You know? It [the SUPER-HEALTH program] gives me a reason to get up and going (ID 448).
Built activity habit	It encouraged me to exercise more and have some accountability for my wellbeing (ID 32). It kind of helped me stay in a routine (ID 188). The routine. It follows a routine, and you follow a routine, and you get into that routine, and there it is (ID 305). What prompted me is, again, I like the set routine, and it was easily navigable. I could navigate the exercise videos and the articles. Reading the articles are wonderful. But just having something to get up and do, know I got to get it done and be done with it. I mean, it's not the social aspect, but it's just the routine and the – I can't think of the... accountability (ID 327).
Improved psychosocial health	
Mood enhancement	It boosted my mood, I guess. Um, it, I, I re- it did make me, like, happier (ID 188). I feel great. I feel energized. I feel like I'm ready for the day. I'm ready to carry through those good habits throughout the day when I exercise (ID 327). I usually feel really good when I'm finished, I mean because they end with the slow stretching and all and cool down. But by the time you're done, you're ready to go [for the day] (ID 334). It just keeps me going because, like I said, I don't want to go back in a wheelchair, and I don't want my heart to give out, and I want to be able to breath, and I want to be healthier. You know, the first part of COVID, I was pretty sedentary, so this is got me going again, and it's making my outlook better for life. Does that make sense? (ID 445)
Sleep quality	I'll just sit there and- and work out and do my- my exercise and it's- it's very good. I feel good, I'm breathing good and, uh, I do it and put it down and go and have a good night's sleep. You know, and it's good. It's very pos- it's very positive. And going over there to SUPER-HEALTH was very positive (ID 166).
Self-efficacy	It [having the watch and stuff and letting me see my progress everyday] made me feel like I was accomplishing things (ID 188). Uh, feel like I've accomplished something (ID 133). I feel really good. It tires me out, but I feel good that I do this, makes me happy that I was able to do it (ID 228). I love about the program is that its people like me doing the exercises and doing the videos. Now, I'm not in a wheelchair, but there's a couple of exercise videos that there is a young man in a wheelchair, and they're not size twos, and they're not wearing these cute little yoga clothes. They're in sweatpants and a t-shirt. They're people like me that are leading the exercise videos. I love that (ID 327). [And how do you feel when you're exercising [with the program]? Pretty good,
Optimized performance and benefits	
Tailor to level of performance	Well, the only the negative thing that I can say is that when you have some of the persons [instructor] that's doing the exercises, they can go quite fast, and I can't keep up. The person have to be mindful that, a person that's ambulatory, we don't have the same trunk function, especially if someone's spinal cord or someone's mobility impaired. We don't have the same function, so their flexibility trying to keep up with the tape has been challenging (ID 32). If you missed a day there was this- this, uh, one person [instructor] that was on there. And she did a really good job and came back the next time and it was- it was a normal speed. And she came back and- and she was thinking you'd remember everything you did the last time when we'd go up a notch. And that was kinda interesting. It was a little pain 'cause not everyone can go as quickly as she was. No, um, I appreciated what she was doing. It was like, wait, I'm not that fast yet, I need to go back to- instead of going from one to three, let's go to two next time if that makes any sense at all (ID 166). Well, some of the moves were pretty advanced for me, or like too fast. So, I'd kind of just like do something different that I could do (ID 188). To be honest with you, I really, I liked it at first, but it was very- the instructor was very hard to follow (ID 198) I was, the exercise routines that, um, it went through were really, um, slow and not very challenging, I guess to me. So, I, I really, I did those for about six weeks, eight weeks-... and then I kind of ended up falling off of doing those (ID 287). For a while, the videos and, um, I kind'a over and over I kind of thought that the videos weren't really like, my speed. I thought they were a little bit slower than what I needed. And they weren't, you know- I'm used to a workout that you can feel, and that is like really difficult, you know, um, and I never got that [the feeling of difficulty/challenge] from those (ID 58).
	I guess when I started to overcome, you know, my flares and I think I could do a little bit more. I wish it was a little bit more options for people that were not like immobile like starting off. I just wish it was just a little bit more options. Like increasing the intensity. I just wish it had been like maybe two different videos for somebody that's like immobile and then somebody that was a little bit more mobile to choose from (ID 379).
Flexibility to customize exercise program	The only thing is they're not sequential. The articles are not sequential, and some of the videos are not. You know, I would think the latest video would be the first one and it's not; it's at the bottom. That's the only thing, and that's just me. It's just an OCD of mine. You know, I would think that the last one would be at the bottom and the new one would be at the top to read, but you have to scroll down and hunt the week that you're looking for (ID 327). There's probably room for improvement as far as accessing and sequencing videos. I would suggest the participant could customize a workout routine, and maybe pick videos five, seven, and nine, for example, for an aerobic routine, and then pick three more videos and be able to link those and just pile them in a group in a given session. I would suggest to you, being able for the participant to link videos to create a user design workout routine. That might be an upgrade I'd like to see (ID 332).
Human support for better tailoring prescription	Uh, some-, you know, I have lower, lower back pain. And then, during, uh, a good portion of the, uh, st-study, I had rotator cuff issues and they were gonna give me a steroid but I, and um, but I just did not want that, you know. Um, and I would of like, you know, a live person sometimes, you know. There could of been someone [like trainer], you know, I, I understand that you know, they just can't just, you know, pro-, you know, provide the man power for everybody who was involved to, um, you know, maybe do it with you or to guide you through it. But, you know, that would of been really, really cool (ID 133). I guess if I had, I don't know, maybe a more, um, personalized approach to a fitness program, maybe that would've tapped into more of my energies. I don't, if that makes any sense? Or create... I don't know, not that, I'm, I'm... I don't know. Well, hmm, it would be great to be able to meet with somebody, um, to teach me, um, proper stretching techniques, to watch me work out with the weights system, to, um, to make sure I'm doing that correctly. Um... and I'm gonna guess obviously to monitor, you know, my heart rate and make sure I'm not overdoing it or... I don't know. I don't think I ever really overdo it obviously or I wouldn't be overweight. Um... I mean, it, it would be, yeah, it would be great to have, I mean, it would be (ID 183). Just one comment about the completion of the program, again, I like human interaction. If I had one thing, my number one suggestion for the program would be to have some opportunity to meet in person with the participants and the leadership. Well, I know you do it by Zoom. I guess I'm old school. I don't think there's any substitute for in-person activity (ID 332).

### Themes

#### Theme 1: Improved physical health and function

The first theme highlighted *perceived benefits on physical health and function* those participants have experienced after the intervention period. Specific benefits included improvements in muscle strength and endurance, flexibility, balance, and walking. Participants reported that they felt more strength in their legs, core, and arms, as well as released muscle/joint stiffness, which further helped them decrease pain and ease the performance of daily activities (e.g., increasing energy level, walking long periods or with less use of cane). The program was also perceived to help reduce weight and increase balance and coordination, such as turning the head around safely while walking or driving.

#### Theme 2: Increased activity participation

Participants stated that they increased the volume of physical activity they performed after joining the program. Contributors to this increase in activity were participation in the exercise prescription and additional activities that they performed outside of the program (e.g., walking the neighborhood, counting steps, and lifting weights while watching TV). Participants stated that SUPER-HEALTH provided the needed knowledge of how to perform physical activity as a person with a disability and a reminder and encouragement to be physically active. Several participants noted how SUPER-HEALTH helped them to adopt a more active lifestyle.

#### Theme 3: Improved psychosocial health

The third theme highlighted that the program helped their *psychosocial aspects of health*. The benefits included mood enhancement, better sleep quality, and increases in self-efficacy. Participants often reported that they felt happier, energized, and mentally sharp after the exercise, which helped them continue the exercise and more activities throughout the day. They also noted enhanced confidence in their physical ability when accomplishing the prescribed exercise sessions and realizing their physical strength and ability with new movements.

#### Theme 4: Optimized performance and benefits

To optimize their performance and benefits, participants reported that the program could be better tailored to their abilities and interests. While many participants commented that the exercises were appropriate for their ability throughout the intervention period, some participants perceived that the program content was not appropriate for their functional ability/level (too challenging or too easy). Participants described challenges in some exercise routines that the movements were too fast and hard to follow, which created feelings of frustration. In contrast, some participants reported that too easy/slow exercises were not perceived as “exercise” and created feelings of boredom and decreased motivation to participate.

Some participants stated a desire for the ability to *design an individualized program*. Some included having the sequence of videos rearranged to increase the motivation and interest (e.g., the newest video on the top). They also suggested emphasizing exercise components meeting their specific needs and health concerns (e.g., focus on flexibility for pain due to tight muscles and focus on cardio for someone who has heart issues).

Some participants desired occasional human connection/support from research staff (instructors, coaches) *via* calls or Zoom meetings. They described that potential follow-up during the intervention can answer frequent questions for exercise programs (e.g., variation/adaptation of exercise difficulty, clarification of exercise movements, and the suggestion of appropriate weight), which could enhance the benefits received from the exercise sessions.

## Discussion

This paper presented participants' perspectives for a randomized controlled trial aimed to investigate the effectiveness of a convenient telehealth exercise program for adults with neurological and physical disabilities. Currently, there are minimal exercise guidelines for PWD, and much more research is needed to develop effective interventions for increasing the benefits of exercise in this population (20, 21). Preliminary findings from this qualitative study indicate the potential benefit of a telehealth physical activity program for PWD. These perceived benefits included improvements in health and function, such as muscle strength and endurance, which translated into increased activity participation. For PWD, reducing secondary health conditions, such as pain and fatigue, is a priority for improving overall health, and several participants stated this as a benefit they received from the program. These benefits are similar to those found in onsite M2M-based programs (22, 23).

Suggestions for future telehealth exercise programs involved the precise tailoring of program support and program content. For program content, this can be completed in a couple of ways, such as delivering exercise routines through live, synchronous sessions, where participants schedule a time with the exercise instructor each week to complete sessions using video-conferencing and real-time monitoring technology. Another option is to allow users to create a ‘user design workout routine', where participants can choose exercise videos and build their routines. This would allow participants to create a program that emphasizes their personal health needs, such as more flexibility, strength, or cardiovascular health. This would also allow participants to target specific health issues they encounter based on disability etiology (e.g., neurological and musculoskeletal) using exercise guidelines targeting a specific disabling condition, if available (24, 25). Program support could be enhanced with the synchronous training, which allows the exercise instructor to provide both instrumental support through instructing movements and modifications to exercise routine and emotional support through verbal encouragement. Another suggestion for tailoring program support is to provide features that allow participants to share exercise schedules with other participants to enable a separate but synchronous exercise. Utilizing current technology for enhancing social connectedness can provide a seamless strategy for participants to organize their synchronous sessions with others.

SUPER-HEALTH was a large study of its kind, which used telehealth technology to encourage sustainable home exercise among underserved and disabled adult populations. The preliminary results of the trial show the effectiveness of this home exercise program, emphasizing the growing need for these convenient physical activity interventions. The exercise program, M2M, does this by making the exercise fun (with music and routines), and subjective to each participant's functional level by tailoring certain exercises and exercise intensities to them. The M2M program is provided in the form of music, which bolsters enjoyment and, therefore, participation, increases future scalability because it is seen as “fun” and can still be modified to be novel (through new music and movement patterns, while keeping the general premise the same). The constant monitoring technology used in this trial allows researchers to gauge when participants are not fully engaged in their exercise programs and prompts for more tailored coaching and adjustments. Importantly, participants stated no issues with utilizing the mobile health application or video delivery. Although this is a small sample of participants from the study, this should provide clinical providers an assurance that the usability of current technology for delivering exercise programs has minimal barriers for patients.

The purpose of the study was to collect qualitative data on participants' perceived benefits of the SUPER-HEALTH program. The study had several limitations. This study was conducted in one region of the United States and the findings may not be generalizable to other regions across the country or in other countries. This was a retrospective evaluation and some of the participants were interviewed at 24 weeks, while others were interviewed further out including up to 6 months from the 24-week endpoint. A purposeful sampling strategy was used to obtain diversity in interviews, which could introduce bias. Lastly, the sample was composed primarily of women (70%) and the findings may not be as representative of males.

## Conclusion

SUPER-HEALTH connected participants to health professionals in the convenience of their home providing accessible exercise routines. Transportation and program costs are two of the most common barriers reported among those with physical disabilities and this program eradicates that issue. Telehealth in this specific study also confluences with current technologies in activity monitoring (FitBit), which allows the participant to receive a more accurate activity monitoring. Future programs should include tailoring of support and program content, such as exercises, coaching support, and program progression.

## Data availability statement

The raw data supporting the conclusions of this article will be made available by the authors, without undue reservation.

## Ethics statement

The studies involving human participants were reviewed and approved by UAB Office of Institutional Review Board. The patients/participants provided their written informed consent to participate in this study.

## Author contributions

JW, NI, and IH developed interview guides. JW was responsible for conducting the interviews. JW and YK analyzed the data. JW, YK, TS, and BL created the initial draft of the manuscript. All authors contributed to the final manuscript draft.

## Funding

Funding for this study was provided by the National Institutes of Health, Eunice Kennedy Shriver National Institute of Child Health and Human Development (5R01HD085186) and a Mentored Career Development Award from the National Center Advancing Translational Research (KL2 TR 003097).

## Conflict of interest

The authors declare that the research was conducted in the absence of any commercial or financial relationships that could be construed as a potential conflict of interest.

## Publisher's note

All claims expressed in this article are solely those of the authors and do not necessarily represent those of their affiliated organizations, or those of the publisher, the editors and the reviewers. Any product that may be evaluated in this article, or claim that may be made by its manufacturer, is not guaranteed or endorsed by the publisher.
